# The Dutch Idiopathic Ventricular Fibrillation Registry: progress report on the quest to identify the unidentifiable

**DOI:** 10.1007/s12471-024-01870-y

**Published:** 2024-04-23

**Authors:** Lisa M. Verheul, Sanne A. Groeneveld, Job Stoks, Wiert F. Hoeksema, Matthijs J. M. Cluitmans, Pieter G. Postema, Arthur A. M. Wilde, Paul G. A. Volders, Rutger J. Hassink

**Affiliations:** 1 Department of Cardiology, University Medical Centre Utrecht, Utrecht, The Netherlands; 2 Department of Cardiology, Cardiovascular Research Institute Maastricht, Maastricht University Medical Centre+, Maastricht, The Netherlands; 3 Department of Cardiology, Amsterdam UMC, location University of Amsterdam, Amsterdam, The Netherlands; 4 Amsterdam Cardiovascular Sciences, Heart Failure and Arrhythmias, Amsterdam, The Netherlands

**Keywords:** Sudden cardiac arrest, Idiopathic ventricular fibrillation, Diagnostics, Follow-up

## Abstract

**Background:**

Idiopathic ventricular fibrillation (iVF) is a rare cause of sudden cardiac arrest and, by definition, a diagnosis of exclusion. Due to the rarity of the disease, previous and current studies are limited by their retrospective design and small patient numbers. Even though the incidence of iVF has declined owing to the identification of new disease entities, an important subgroup of patients remains.

**Aim:**

To expand the existing Dutch iVF Registry into a large nationwide cohort of patients initially diagnosed with iVF, to reveal the underlying cause of iVF in these patients, and to improve arrhythmia management.

**Methods:**

The Dutch iVF Registry includes sudden cardiac arrest survivors with an initial diagnosis of iVF. Clinical data and outcomes are collected. Outcomes include subsequent detection of a diagnosis other than ‘idiopathic’, arrhythmia recurrence and death. Non-invasive electrocardiographic imaging is used to investigate electropathological substrates and triggers of VF.

**Results:**

To date, 432 patients have been included in the registry (median age at event 40 years (interquartile range 28–52)), 61% male. During a median follow-up of 6 (2–12) years, 38 patients (9%) received a diagnosis other than ‘idiopathic’. Eleven iVF patients were characterised with electrocardiographic imaging.

**Conclusion:**

The Dutch iVF Registry is currently the largest of its kind worldwide. In this heterogeneous population of index patients, we aim to identify common functional denominators associated with iVF. With the implementation of non-invasive electrocardiographic imaging and other diagnostic modalities (e.g. echocardiographic deformation, cardiac magnetic resonance), we advance the possibilities to reveal pro-fibrillatory substrates.

**Supplementary Information:**

The online version of this article (10.1007/s12471-024-01870-y) contains supplementary material, which is available to authorized users.

## What’s new?

- The Dutch Idiopathic Ventricular Fibrillation Registry is one of the largest national cohorts with patients initially diagnosed with idiopathic ventricular fibrillation.
- During follow-up, 9% received a diagnosis other than ‘idiopathic’ and 25% received appropriate implantable cardioverter defibrillator therapy.
- Non-invasive electrocardiographic imaging and other diagnostic modalities are used to reveal underlying substrates.

## Introduction

**Fig. 1:**
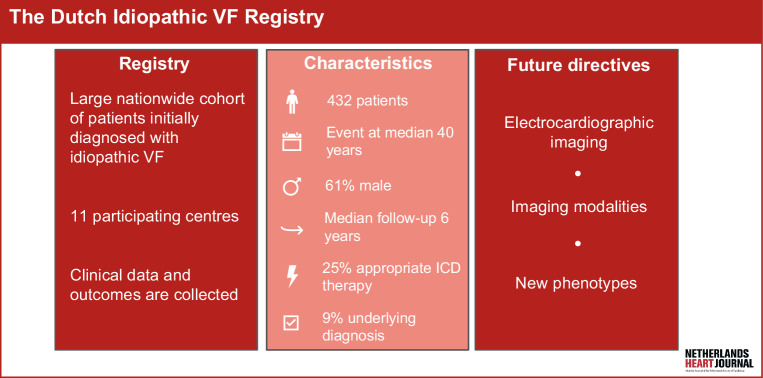
Infographic: the Dutch Idiopathic Ventricular Fibrillation Registry. *VF* ventricular fibrillation, *ICD* implantable cardioverter defibrillator

When all identifiable causes of ventricular fibrillation (VF) are excluded with systematic diagnostic testing, a patient with sudden cardiac arrest (SCA) due to VF is diagnosed as “idiopathic” (iVF) [1]. iVF is a rare diagnosis, and approximately 2–7% of all patients presenting with SCA are considered idiopathic [2, 3]. Over the years, the incidence has declined due to identification of new disease entities. For example, with the ongoing improvement of imaging techniques, structural abnormalities are more often recognised [4]. Newly discovered disease entities mainly include primary arrhythmia syndromes, such as Brugada syndrome [1]. Complete diagnostic testing is considered the cornerstone in iVF, as shown by our latest registry publication. However, such testing appears not to have been performed consistently in studies to date [5, 6]. Moreover, the electrophysiological mechanisms leading to VF in these patients are still unidentified. Several factors, such as the heterogeneity of the iVF population, are hampering this identification. To enlarge our knowledge about iVF, the Dutch iVF Registry was designed, creating a large national, multicentre cohort (Fig. 1: Infographic). This progress report focuses on following up on our first report of the registry in 2018 and highlights the output over the years [7].

## Methods

### Objectives

The need for the Dutch iVF Registry was stated in our first report, in which we introduced the registry in the *Netherlands Heart Journal* [7]. With the registry we aim to (1) create a large cohort of patients with an initial iVF diagnosis, (2) focus on additional diagnostic testing (e.g. deformation echocardiography, cardiac magnetic resonance (CMR) and electrocardiographic imaging (ECGI)) to reveal a possible substrate, and (3) collect data on the long-term follow-up, including genetic testing, implantable cardioverter defibrillator (ICD) therapy and family screening.

### Study population

**Fig. 2:**
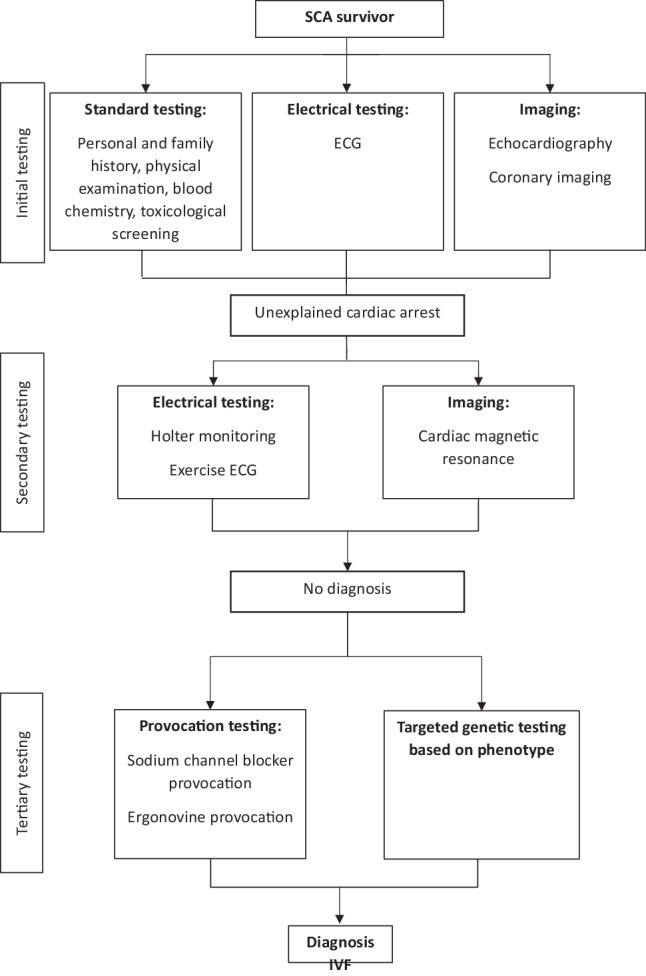
Diagnostic approach after a sudden cardiac arrest (*SCA*). Flowchart indicating all diagnostic tests that need to be performed before diagnosing a patient with idiopathic ventricular fibrillation (iVF). After first investigation with standard testing (electrical testing with an electrocardiogram (ECG) and imaging testing including both echocardiography and coronary imaging) a patient is considered an unexplained cardiac arrest survivor. After additional testing, the diagnosis of iVF can be made. Adapted from Visser et al. [1], with permission from Wolters Kluwer Health, Inc

Eligible patients are those with documented VF, cardiac arrest with a shockable rhythm, or sustained polymorphic ventricular tachycardia (VT), for which known cardiac, respiratory, metabolic and toxicological aetiologies are excluded, as defined by the latest consensus criteria [8]. Preferably, a specific diagnostic approach is followed before diagnosing a patient with iVF. A flowchart indicating which diagnostic tests should be performed at a minimum before diagnosing a patient with iVF is shown in Fig. 2; [1, 4]. If diagnostic test results reveal minor abnormalities, insufficient for a specific diagnosis, this is not an exclusion criterion. Before inclusion in the registry a minimum of 50% of the listed diagnostic tests need to be performed.

### Data collection

Data are collected from electronic health records both retrospectively and prospectively, following a standardised form using Castor EDC [9]. We obtain the following data: medical history, physical examination, laboratory testing (including toxicological screening), electrical testing (electrocardiogram (ECG), Holter monitoring, exercise treadmill test (ETT), electrophysiological study), imaging data (echocardiography, coronary imaging, CMR, cardiac computed tomography, positron emission tomography), provocation testing (sodium channel blocker provocation (SCBP), coronary artery spasm provocation), DNA analysis and endomyocardial biopsy results. We consider a diagnostic work-up optimal when an ECG, Holter/telemetry, ETT, echocardiography, CMR, coronary imaging and SCBP are performed. Based on the literature, high-yield diagnostic tests are an ETT, CMR and SCBP [5, 6]. During follow-up, we advise local physicians to complete the diagnostic work-up of iVF patients to reach the most optimal diagnostic work-up in all patients. Outcomes are collected at baseline and during follow-up. Outcomes include appropriate ICD therapy (antitachycardia pacing or shock), inappropriate ICD therapy, detection of a diagnosis, and death. We collect follow-up data every 1–2 years in each participating centre.

### Electrocardiographic imaging

The Dutch iVF Registry is incorporated in the VIGILANCE consortium (CVON project). VIGILANCE is an acronym for ‘non-in**v**as**i**ve electrocardio**g**raph**i**c imaging for individua**l**s **a**t risk for appare**n**tly idiopathi**c** v**e**ntricular fibrillation’, indicating the fundamental scientific role of ECGI in our research. ECGI enables the non-invasive measurement of the electrical activity at the level of the heart with the use of body surface ECGs and a specific heart-torso geometry [10].

For ECGI measurements and analysis, previously described methods were repeated [11]. In short, approximately184 electrodes were placed on the patient’s torso. Body surface potentials were recorded for 10–60 min. Cardiac computed tomography was used to acquire the heart geometry during end-diastole using intravenous iodine contrast; thoracic computed tomography was used for the positions of the body surface electrodes. This allowed the approximation of the electrostatic torso-heart relationship. Afterwards, for each subject, epicardial electrograms were non-invasively reconstructed for a sinus beat, and each morphologically distinguished premature ventricular contraction (PVC) when applicable. The activation time of each epicardial node was determined by the steepest downslope of the QRS complex; repolarisation time was determined by the steepest upslope of the T‑wave [12].

## Results

As of 1 December 2022, the Dutch iVF Registry contained 432 patients from 11 different hospitals across the Netherlands. An overview of the patients’ clinical characteristics is listed in Tab. 1. The performed diagnostic work-up is presented in Tab. 2. For almost all patients, results from an ECG, ischaemia detection and one imaging modality were available. In 175 patients (41%) all three high-yield diagnostic tests were performed. All patients had a median follow-up of 6 (2–12) years. In total, 38 patients (9%) received a diagnosis during 11 years of follow-up; 394 patients (91%) remained idiopathic during a median follow-up of 6 years.

**Table 1:** Clinical characteristics of patients included in the registry with an initial diagnosis of idiopathic ventricular fibrillation. High-yield diagnostic tests include exercise treadmill test, cardiac magnetic resonance and sodium channel blocker provocation

	All	Idiopathic	Diagnosis
Total	432 (100%)	394 (91%)	38 (9%)
Age at index event (years)^a^	40 (28–52)	40 (29–52)	38 (26–54)
Male sex	262 (61%)	237 (60%)	25 (66%)
Family history of SCD	67/424 (16%)	59/388 (15%)	8/36 (22%)
Follow-up duration (years)^a^	6 (2–12)	6 (2–12)	11 (6–17)
Follow-up available > 1 year	345 (80%)	310 (79%)	35 (92%)

*SCD* sudden cardiac death

^a^Data are provided as median (IQR)

**Table 2:** Diagnostic work-up performed in patients initially diagnosed with idiopathic ventricular fibrillation. High-yield diagnostic tests include exercise treadmill test, cardiac magnetic resonance and sodium channel blocker provocation

	Diagnostic work-up^a^
Electrocardiogram	432 (100%)
Toxicological screening	66 (15%)
Holter telemetry	281 (65%)
Echocardiography	423 (98%)
Exercise treadmill test	306 (71%)
Coronary imaging	404 (94%)
Cardiac magnetic resonance	323 (75%)
Sodium channel blocker provocation	274 (63%)
Ergonovine provocation	80 (19%)
Genetic testing	376 (87%)
All high-yield diagnostic tests performed	175 (41%)

^a^In some patients diagnostic tests could either not be performed or relevant data were missing

### Clinical course

The clinical course of patients is summarised in Fig. S1 in Electronic Supplementary Material (ESM). Most patients presented with an arrhythmia occurring at rest (*n* = 253, 59%). An ICD was implanted in 383 of 394 iVF patients (97%) and 35 of 38 patients (92%) who received a diagnosis during follow-up. Reasons for not undergoing ICD implantation varied among iVF patients: 4 patients refused implantation, 2 patients had an event prior to ICD availability, 2 patients underwent successful quinidine treatment, 1 patient had polymorphic VT, and 1 patient exhibited impaired cognitive status. Among patients who remained idiopathic, 100 (25%) had ventricular arrhythmia (VA) recurrence resulting in appropriate ICD therapy. When focusing on the first recurrence, most cases occurred at rest. The underlying VA was VF in the majority (65% with VF, 15% with VT and 16% with both VF and VT). Among patients with available registrations of an initiation during follow-up (*n* = 58), VAs were initiated by a PVC in 42 (72%). Short-coupled initiations (coupling interval < 350 ms) were present in 36 patients. In total, 19 patients died during follow-up, 7 of them due to a cardiac cause. Three of the patients who died due to a cardiac cause had remained idiopathic (3/394, 1%) and 4 patients (4/38, 11%) had received a diagnosis during follow-up. VAs were the (presumable) cause in 4 patients, of which 2 did not receive ICD implantation. The remaining 3 patients died due to end-stage heart failure.

### Diagnosis

Specific diagnoses are further specified in Tab. 3. Most patients were diagnosed with either a cardiomyopathy (47%) or channelopathy (37%). In 2018, all ECGs were re-evaluated to diagnose early repolarisation syndrome (ERS), after ERS was identified as a distinct phenotype in iVF patients [13]. Non-diagnostic findings on echocardiography and/or CMR during the first event were prevalent in patients eventually diagnosed with a cardiomyopathy. Patients with VT recurrence (*n* = 31) did not receive a diagnosis during follow-up. For 3 patients diagnosed with Brugada syndrome, provocation testing was not initiated during baseline diagnostic work-up. Performing this test during follow-up revealed a type‑1 Brugada ECG pattern and the diagnosis was made. The *DPP6* risk haplotype was found in 37 patients (9%). This Dutch founder risk haplotype located on chromosome 7q36 is associated with iVF and has been characterised previously in detail [14, 15]. Since the specific mechanism causing VF in these patients remains unknown, they are still considered idiopathic.

**Table 3:** Overview of diagnoses

Diagnosis	Number of patients	Findings before diagnosis
Total	38 (100%)	
Cardiomyopathy	18 (47%)	
– Arrhythmogenic cardiomyopathy	9 (24%)	9 patients with non-diagnostic findings at baseline (100%)
– Dilated cardiomyopathy	5 (13%)	2 patients with non-diagnostic findings at baseline (40%)
– Hypertrophic cardiomyopathy	4 (10%)	4 patients with non-diagnostic findings at baseline (100%)
Channelopathy	14 (37%)	
– Brugada syndrome	5 (13%)	3 patients without provocation test at baseline, resulting in a diagnosis during follow-up (60%)
– Long-QT syndrome	1 (3%)	
– Catecholaminergic—polymorphic VT	5 (13%)	5 patients with ectopy during diagnostic testing at baseline (100%)
– Early repolarisation syndrome	3 (8%)	
Myocarditis	1 (3%)	
Coronary artery spasm	2 (5%)	
Other	3 (8%)	

*VT* ventricular tachycardia

### Electrocardiographic imaging

First ECGI results with iVF patients were published in 2021 [16]. Eleven iVF patients were included. The registry enabled expansion of ECGI measurements to a large patient cohort, involving both patients (*n* = 59) and their family members (*n* = 13). We expect the first results of these measurements to be available soon.

## Discussion

The Dutch iVF Registry provides the opportunity to investigate a large population of iVF patients. With the growing registry population, several previous knowledge gaps have now become clearer.

### Follow-up and diagnostic work-up

In 2016 already, a first report on only a small subset (107 patients) of the current registry provided information about the number of diagnoses and appropriate ICD therapy during follow-up [17]. By enlarging the registry, this information has become more accurate and now shows a diagnostic rate of 9% (compared to 21% in 2016) and 26% of patients having appropriate ICD therapy (compared to 29% in 2016) during follow-up [6, 17]. The absolute number of patients with an alternative diagnosis increased from 22 patients in the 2016 report to 38 patients in this present report. However, by enlarging our registry the median follow-up decreased. Therefore, concealed diseases might still need to unfold in time. In 2018, the focus was on ICD therapy in iVF patients [18]. Besides appropriate ICD therapy, complications and inappropriate shocks were frequent (14.7 and 17.5%, respectively). Nevertheless, ICD implantation remains the only ‘treatment’ for iVF overall. Specifically for short-coupled iVF, quinidine appears to be the preferred treatment [19]. Due to the unavailability of quinidine in several countries, optimising this treatment has been a challenge. Our latest registry update focuses on the value of the diagnostic work-up [6]. Just recently, an expert consensus statement was published recommending a specific diagnostic approach in SCA survivors [20]. An initial diagnostic work-up of SCA survivors with an ECG, echocardiography and coronary angiography results in a diagnosis in approximately 88% of patients [2]. After this, high-yield tests are important to reveal additional diagnoses [6].

### The search for a substrate with new diagnostic techniques

#### Imaging modalities

We investigated the use of echocardiographic deformation imaging in patients diagnosed with iVF, which is a modality known for its ability to reveal concealed disease stages [21, 22]. Both global and regional echocardiographic deformation abnormalities were more prevalent in iVF patients compared to age- and sex-matched control patients. The main difference between these groups was the occurrence of a circulatory arrest in iVF patients. It remains undetermined if the abnormalities found were caused by the arrest itself (and global hypoxaemia during the arrest) or are evidence of a concealed cardiomyopathy [21]. Another focus has been on mitral annulus disjunction (MAD) and mitral valve prolapse. Primarily MAD has been gaining attention in recent years and a possible arrhythmogenicity has been investigated. Zugwitz et al. showed with the use of the UK Biobank that the prevalence of MAD is in general high [23]. However, inferolateral MAD was found to be uncommon, and it appeared that specifically inferolateral MAD was more prevalent in iVF patients compared to a control group [24]. This finding justifies further validation.

#### Electrocardiographic imaging

**Fig. 3:**
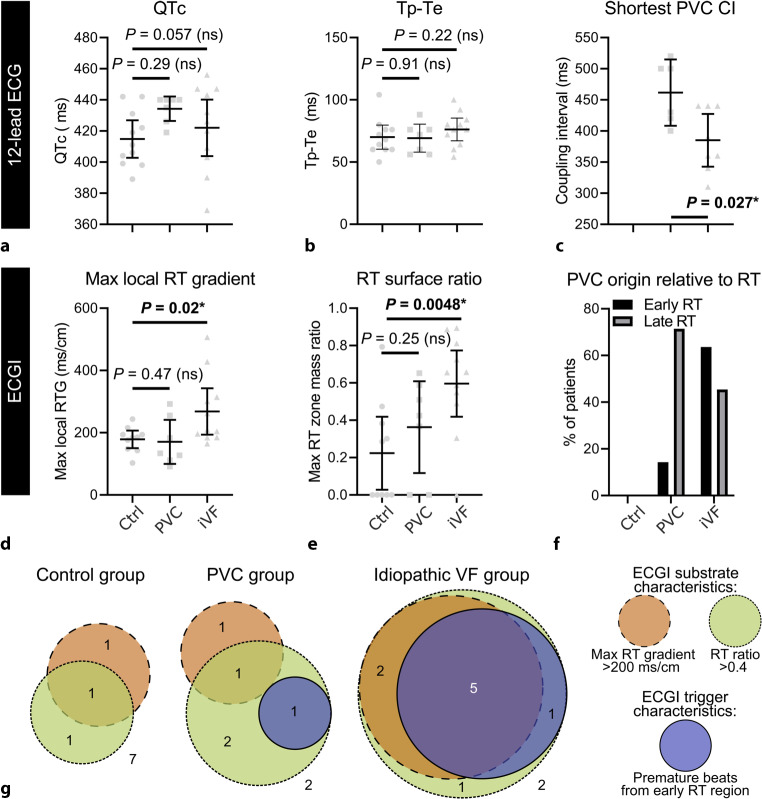
**a**–**g** Electrocardiographic imaging (*ECGI*) characteristics stratified between a control group (*Ctrl*), premature ventricular contraction (*PVC*) group and idiopathic ventricular fibrillation (*iVF*) patients. **a–c** Conventional 12-lead ECG results of repolarisation (QTc and *T*_peak_-*T*_end_ interval) and PVC coupling interval. **d–f** ECGI results. **g** The repolarisation substrate characteristics (maximal RT gradient, RT ratio) and ECGI trigger characteristic (PVC region), stratified by control, PVC and iVF patients. *RT* repolarisation time, *RTG* repolarisation time gradient, *VF* ventricular fibrillation. From Cluitmans et al. [16]. Reprinted with permission from the American Association for the Advancement of Science

Besides additional imaging techniques, we focused on the use of (epicardial) ECGI. In the study by Cluitmans et al., a repolarisation substrate facilitating re-entry in iVF patients is presented. A repolarisation substrate depends on at least four abnormalities to facilitate re-entry [16]. First, regions of early and late repolarisation with steep repolarisation time gradients in between need to be present. Second, the sizes of these regions should be balanced. Third, a PVC should arise in an early repolarising region. This PVC will block and cannot activate a region with late repolarisation, as this is not yet excitable, However, when the early repolarisation region is large enough, the PVC can initiate re-entry when the late repolarisation region becomes repolarised. Last, this PVC should be critically timed to initiate re-entry. The described characteristics were more prevalent in iVF patients, as illustrated in Fig. 3 [16]. Conventional 12-lead ECG measurements for repolarisation (Fig. 3a, b) were not significantly different between the investigated groups. However, ECGI revealed that repolarisation time in iVF patients was more heterogeneous, with steeper repolarisation time gradients and relatively large early repolarisation regions (Fig. 3d, e). PVCs in iVF patients more often arose from early repolarisation regions (Fig. 3f) and had a shorter coupling interval on conventional 12-lead ECG (Fig. 3c). Figure 3g shows the repolarisation substrate characteristics present in each group [16]. This study provides evidence for repolarisation abnormalities that might form a substrate to induce iVF. As only a small subgroup of the iVF patients were characterised using ECGI, we will next focus on extending these measurements.

### Future perspectives

We will continuously expand the registry and intend to include all ICD implanting centres in the Netherlands. Newly diagnosed patients are eligible for inclusion. Currently, the registry is limited by its observational and retrospective nature, associated with missing data. However, as the foundation of the registry is now established, we can further focus on the prospective part of the registry, complete our missing data, and expand our follow-up. In iVF, the follow-up is of utter importance. Only during follow-up can a concealed or new disease entity be revealed [25]. Interestingly, based on our results and corroborated by others, a VT is also found as the underlying rhythm during recurrence of VAs. This should trigger the initiation of new diagnostic tests. Future studies are needed to provide more guidance on performing diagnostic tests during follow-up [26, 27]. Furthermore, a possible new disease entity in iVF is short-coupled VF. The high prevalence rate of 91% in our previous report makes it debatable whether short-coupled VF might be the true phenotype in iVF [28]. Future research should further define this phenotype.

## Conclusion

The Dutch iVF Registry has made it possible to investigate a large population diagnosed with iVF.. A complete initial diagnostic work-up and re-evaluation during follow-up is important to reveal an underlying disease. By continuously improving our knowledge and incorporating new diagnostic techniques, we are working on exposing all substrates in iVF patients.

### Supplementary Information

Supplement 1: Visualisation of the presentation, clinical course and outcome of 432 initial idiopathic ventricular fibrillation (iVF) patients.
